# Human Immune Responses to Adeno-Associated Virus (AAV) Vectors

**DOI:** 10.3389/fimmu.2020.00670

**Published:** 2020-04-17

**Authors:** Giuseppe Ronzitti, David-Alexandre Gross, Federico Mingozzi

**Affiliations:** ^1^INTEGRARE, Genethon, Inserm, Univ Evry, Université Paris-Saclay, Evry, France; ^2^Spark Therapeutics, Philadelphia, PA, United States

**Keywords:** AAV vectors, gene therapy, T cells, B cells, clinical trials

## Abstract

Recombinant adeno-associated virus (rAAV) vectors are one of the most promising *in vivo* gene delivery tools. Several features make rAAV vectors an ideal platform for gene transfer. However, the high homology with the parental wild-type virus, which often infects humans, poses limitations in terms of immune responses associated with this vector platform. Both humoral and cell-mediated immunity to wild-type AAV have been documented in healthy donors, and, at least in the case of anti-AAV antibodies, have been shown to have a potentially high impact on the outcome of gene transfer. While several factors can contribute to the overall immunogenicity of rAAV vectors, vector design and the total vector dose appear to be responsible of immune-mediated toxicities. While preclinical models have been less than ideal in predicting the outcome of gene transfer in humans, the current preclinical body of evidence clearly demonstrates that rAAV vectors can trigger both innate and adaptive immune responses. Data gathered from clinical trials offers key learnings on the immunogenicity of AAV vectors, highlighting challenges as well as the potential strategies that could help unlock the full therapeutic potential of *in vivo* gene transfer.

## Introduction

Adeno-associated virus (AAV) is a small (25 nm), non-enveloped virus composed by an icosahedral capsid that contains a single-stranded, 4.7-Kb DNA genome. AAVs are *Dependovirus*, as they replicate only in the presence of helper viruses such as adenovirus, herpes virus, human papillomavirus and vaccinia virus (1–4). AAV genome is composed by two genes *rep* and *cap*, flanked by two palindromic inverted terminal repeats (ITR). *Rep* encodes for proteins involved in replication of the viral DNA, packaging of AAV genomes, and viral genome integration in the host DNA (5). *Cap* encodes for the three proteins that form the capsid, VP1, 2 and 3, and for the assembly activating protein (AAP) and the newly identified MAAP (5, 6). Wild-type AAVs naturally infect humans around 1 to 3 years of age (7–9) and are not associated with any known disease or illness (10). After infection, AAV remains latent in integrated or not-integrated forms, until a helper virus provides the functions necessary for its replication (5). In recombinant AAV vectors (rAAV), the parental virus *rep* and *cap* genes are replaced with the DNA of choice flanked by the two ITRs, and referred to as the transgene expression cassette when used for gene therapy purposes. rAAV vectors are produced efficiently by several approaches: transient double or triple transfection of mammalian cells (11, 12); infection of mammalian cell lines with adenovirus (13) or herpes simplex virus (14, 15); and infection of insect cells with baculovirus (16). During packaging, *rep* and *cap* genes areprovided in *trans* together with the adenoviral helper proteins required for AAV genome replication and packaging (17, 18). Triple transfection of HEK293 cells is one of the most commonly used methods for rAAV production. It is based on the co-transfection of three plasmids: one containing the transgene expression cassette flanked by the viral ITRs, a second packaging plasmid expressing the *rep* and *cap* genes and a third plasmid encoding for adenoviral helper genes (17, 19). Historically, the purification of rAAV vectors was performed by ultracentrifugation in two successive density gradients (17). Nowadays, the purification of AAV capsids by affinity chromatography is more frequently used as column process is more scalable and yields high purity preparations that are amenable for clinical use (20). Based on the purification method, rAAV preparations differ in terms of contaminants and the ratio of empty to full capsids. An important focus in the field is the continuous improvement of the rAAV manufacturing processes to increase vector yields and purity while reducing costs (17, 18, 21, 22). A significant concern related to the methods of production and purification is the impact of rAAV purity on the overall vector immunogenicity profile. One obvious example of contaminant is the presence of empty capsid in rAAV preparations (23).

**FIGURE 1:**
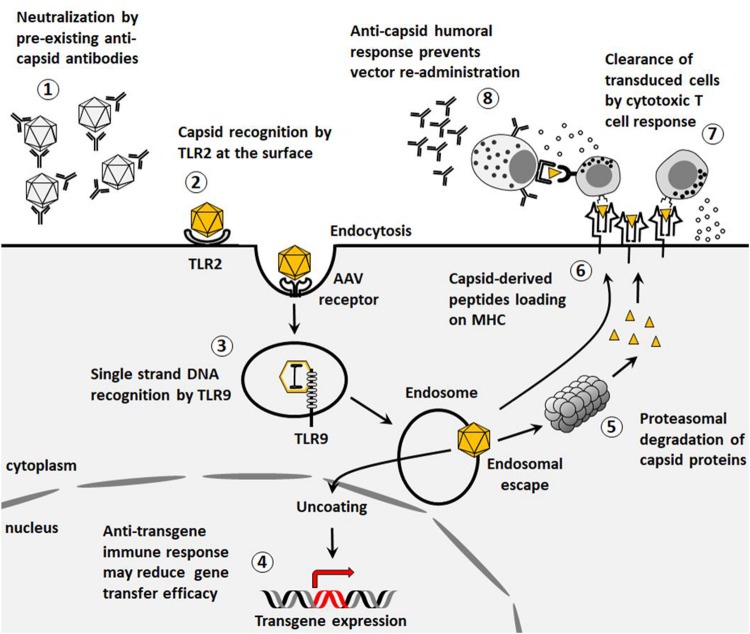
Immunological barriers to gene transfer. **(1)** Pre-existing neutralizing antibodies to AAV vectors reduce gene transfer efficacy. **(2)** Capsids can be recognized by TLR2 at the surface of the cells thus triggering innate immune responses. **(3)** After endocytosis, the viral genome can stimulate TLR9-mediated innate immunity. **(4)** Transgene expression may be associated to the development of an immune response that impacts the outcome of the gene therapy. **(5)** Capsid proteins can be degraded by proteasome and presented on MHC class I. **(6)** Capsid proteins can be presented on MHC class II. **(7)** After presentation on MHC class I, capsid-specific cytotoxic CD8^+^ T cells can clear transduced cells. **(8)** After presentation on MHC class II, anti-capsid humoral response prevents further vector re-administration.

The protein capsid of rAAV affects the affinity of the vector for a given tissue. Transduction of a cell by rAAV vectors requires the interaction of the viral capsid with surface receptors followed by internalization and intracellular trafficking through the endocytic/proteasomal compartment. Capsid proteins then mediate the endosomal escape and nuclear import, and after uncoating, the single stranded genome carried by rAAV is converted to a double stranded DNA. This conversion step may represent a limiting factor to gene transfer that self-complementary (sc) AAV vectors could bypass at the cost of reduced packaging capacity (24). Different from wild-type AAV, the genome of rAAV vectors inefficiently integrates into the host DNA and remains mostly episomal (10, 25, 26). Transgene expression finally results from the transcription of the mRNA and the successive translation of the transgene coding sequence (Figure 1) (27).

To date, 13 different AAV serotypes and 108 isolates (serovars) have been identified and classified (5, 28). The relatively low complexity of AAV biology facilitates production of rAAV vectors composed by a transgene expression cassette flanked by the ITRs from serotype 2 (29) pseudotyped into any of the available AAV capsid variants (5). This process allows a comparison of the properties conferred to rAAVs by the capsid proteins e.g., tissue targeting, potency or immunogenicity. In particular, the capsid composition influences the first steps of transduction i.e., interactions with receptors on target cells and impact, post-entry trafficking and endosomal escape. Therefore, rAAV vectors bearing different capsids have different transduction potential, but also potentially different immunological properties (5, 30). In recent years, several approaches have been used to enable the generation of engineered rAAV vectors and a significant expansion of the rAAV vector toolkit (31–34). While the increasing knowledge on rAAV capsid structure-function (35) led to the generation of capsid variants by direct mutagenesis of specific amino acid residues, the development of rAAV capsid libraries and high-throughput screening methods resulted in the generation of a variety of novel capsids by directed evolution (31). Recently, two different approaches were reported to overcome the limitations of “conventional” methods of vector evolution. Both methods take advantage of the latest advancements in DNA synthesis and sequencing, and are aimed at the reduction of the complexity of the initial library to be screened either by artificial intelligence (6) or by grafting peptides derived from a subset of proteins involved in specific cellular functions (36). Regardless of the method used, the isolation of new rAAV vector capsids responds to a precise need of increased transduction efficacy with optimized biodistribution and reduced immunogenicity, at least in terms of cross-reactivity with pre-existing antibodies.

**FIGURE 2:**
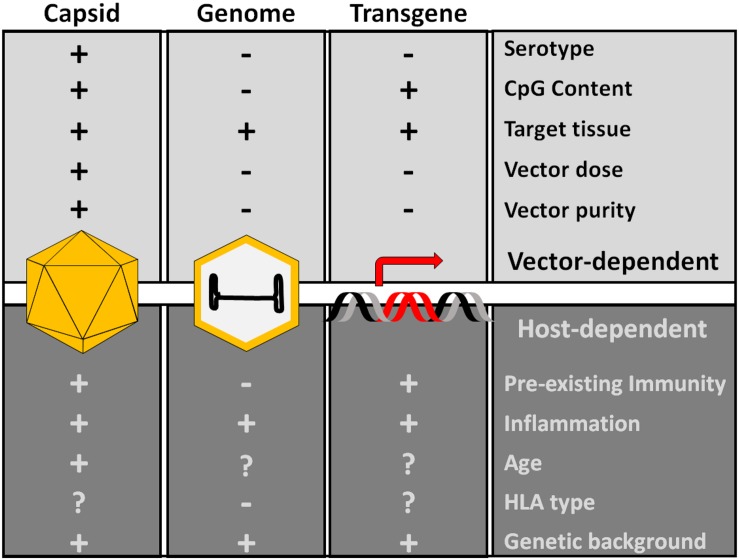
Factors influencing AAV capsid immunogenicity. The proteins of the capsid, the genome and the transgene product are the main potential immunogenic components of AAV vectors. Production of dsRNA driven by the promoter activity of ITRs can also trigger innate immunity. Additional host-dependent and vector-dependent factors can modulate the overall vector immunogenicity. These factors are mostly poorly understood, although innate immunity activators like CpG and vector dose appear to be important determinants of AAV vector immunogenicity.

Despite the efforts dedicated to the enhancement of transduction efficacy, several challenges for clinical use of rAAV vectors remain. Among them, vector immunogenicity, which reflects the interactions of rAAVs with the host immune system, is perhaps of the utmost relevance given its impact on the outcomes of treatment in terms of transgene expression durability, and to the ability to eventually re-administer the vector in case of loss of efficacy over time (Figure 2). Here, we will outline general concept on rAAV vector immunogenicity, and comprehensively discuss the clinical experience in the context of systemic vector administration for liver-targeted gene therapy trials for hemophilia and for the treatment of neuromuscular diseases.

## Human Immune Responses to Wild-Type AAV

### Humoral Immunity to AAV

Although AAV seroprevalence varies geographically, neutralizing antibodies (NAbs) that recognize virtually all AAV serotypes can be found in a large proportion of the human population (ranging from 30 to 60%). Successive infections may explain the high prevalence of anti-AAV NAbs in humans (37–39), resulting in broad cross-reactivity across AAV serotypes of different origins, including human, mammalian or engineered. Regardless of the geographic region, the most prevalent NAbs are directed against AAV2, followed by AAV1 (38). NAbs appear early in childhood with a peak at 3 years of age, leaving a short time-window that is potentially convenient for gene transfer between 7 and 11 months, after the infant loses humoral protection by passive transfer of maternal antibodies (38).

While production of Abs from all four IgG subclasses have been observed, IgG1 is the predominant subclass found in seropositive individuals and, in general, titers of anti-AAV IgG antibodies correlate with the those of anti-AAV neutralizing antibodies (40, 41). Similarly, subjects undergoing gene transfer with AAV vectors develop anti-AAV antibodies from all the four IgG subclasses (mainly IgG1 but also IgG2 and IgG3) as well as IgM, concurring to the resulting high neutralizing titers (42). Interestingly, some individuals carry non-neutralizing IgG binding to the AAV capsid (43). While even low-titer NAbs are associated with efficient vector neutralization *in vivo* (44–46), the presence of binding non-NAbs appear to enhance rAAV transduction efficacy, at least in some tissues (43). Pre-existing anti-AAV antibodies in individuals receiving rAAV vectors are being investigated as a potential source of toxicity related to complement activation (47) although a direct interaction of  rAAV vectors with complement proteins was also reported (48, 49).

### T Cell Responses to AAV

Early clinical trials of gene transfer with rAAV demonstrated the potential negative impact of T cell-mediated immunity on the outcome of gene transfer (45, 50, 51). Since then, research efforts have been focused on the study of pre-existing anti-AAV cell response and on the development of methods to detect and monitor cell-mediated immunity in AAV-based gene transfer (52–55).

Different assays were developed to estimate the frequency of T cells specific for AAV in humans, including IFN-γ ELISPOT (56–58) and flow cytometry based assays (52, 56, 58). In general, AAV-specific cellular responses are less frequently observed than humoral responses, probably due to the lower assay sensitivity and to the fact that capsid-reactive lymphocytes are found in low frequency in peripheral blood. This may explain why different independent studies reported a lack of correlation between pre-existing cellular and humoral immune responses (50, 56, 58). Indeed, efficient AAV-specific T cells detection in peripheral blood and spleen requires several rounds of *in vitro* expansion with peptide libraries derived from the capsid VP1 (50, 56). Alternatively, FACS staining after an AAV specific tetramer-mediated magnetic enrichment can be used to increase the detection of AAV-specific T cells (59). Recently, we showed a correlation between anti-AAV antibodies and circulating AAV2-specific memory CD8^+^ T cells secreting TNF-α (52), suggesting that IFN-γ, which is currently broadly used as a marker of capsid-specific T cell activation, may not be the only cytokine that needs to be tracked for immunomonitoring in gene transfer trials.

As observed with humoral responses, capsid T cell responses are less frequent in young children (<5 years) compared to older healthy donors (56, 60), suggesting that anti-AAV immune responses may arise during infancy after AAV infection, and persist throughout lifetime as a pool of memory T cells in secondary lymphoid organs. Consistently, differentiation markers measured at single cell level by flow cytometry indicated that the majority of AAV-specific T cells found in humans present a memory phenotype (50, 52, 53, 58). After exposure to the capsid antigen, AAV-specific memory T cells produce IFN-γ, IL-2 and TNF-α, and acquire a cytotoxic phenotype measured by the expression of granzyme B and CD107a degranulation markers (52, 53, 56, 58). In addition to that, two patterns of cellular responses to AAV that are dependent on the serology status of patients were recently identified by our laboratory (61). Exposure of human peripheral blood mononuclear cells (PBMCs) obtained from AAV-seropositive donors to capsid epitopes induced an effector memory phenotype in activated CD8^+^ T cells, with secretion of TNF-α, and expression of granzyme B and CD107a (52). In seronegative patients, transient activation of Natural Killer (NK) cells, but not naive CD8^+^ T cells, was observed. The role of these activated NK cells, which secrete both IFN-γ and TNF-α without a cytotoxic phenotype, in the context of gene transfer remains unknown.

## Post-Treatment Immune Responses Against AAV Vectors

### Innate Immunity of rAAV

**FIGURE 3:**
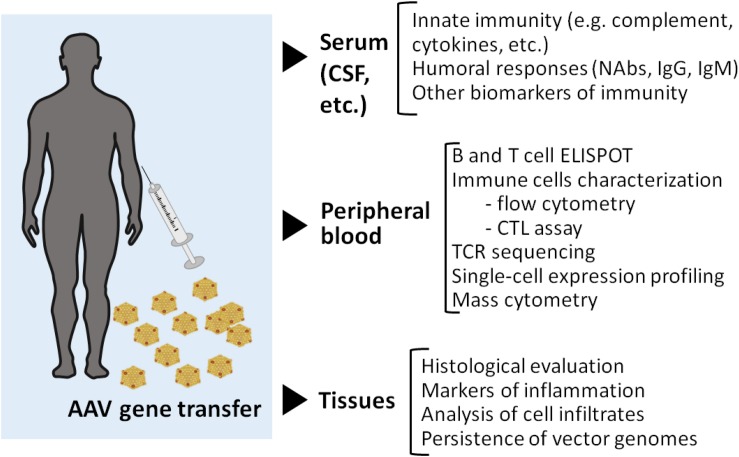
Immunomonitoring in gene transfer. A broad range of assays can be implemented for immunomonitoring in gene transfer trials. Serum samples or other relevant samples like cerebrospinal fluid (CSF) can be used to monitor markers of innate immunity as well as to determine antibody titers before and after vector administration. The cell fraction of peripheral blood is frequently used for both B and T cell assays by ELISPOT. More complex technologies can also be useful for example to track T cell clones via TCR sequencing, or to define transcriptome changes at the single cell level. Additionally, high content flow-based assay can be applied for the simultaneous characterization of a large number of surface and intracellular markers. While a lot of information can be gathered by studying immune response to AAV in peripheral blood, access to tissue samples could potentially help better define the nature of the local immune response in a transduced tissue as well as its impact on vector genome persistence. As many questions remain on AAV immunogenicity, the field of AAV gene therapy research needs further efforts to resolve the complexity of capsid-related immune responses. The harmonization of patient immunomonitoring using standard guidelines, and quality controls to check immune assay performance over time and across clinical trials, would greatly facilitate the comparison of data, and subsequently the understanding of the complexity of anti-AAV immune responses.

Vectors derived from AAV are constituted by a protein capsid, which is highly similar, if not identical, to that of wild type AAV, a single or double stranded DNA genome that does not express any viral proteins, and the inverted terminal repeats (ITR), GC-rich regions of the single stranded genome with a complex secondary structure. Both the capsid and the DNA components of rAAV may concur in the activation of innate immunity along with other host-specific factors (Figures 2, 3). In addition to that, production and purification of the vectors lead to the presence of DNA-depleted AAV capsids (empty capsids) and both DNA and protein contaminants. In comparison to other biological drugs such as monoclonal antibodies, rAAV are quite complex and the prediction of the immune-mediated toxicities after vector administration remains elusive, partially because of the lack of fully predictive animal models (62–64).

In recent years, significant research efforts were focused on establishing a causative role of innate immunity in the immune-mediated toxicities observed in humans. However, the intrinsic characteristics of the innate immune system, together, and the lack of clinical evidence of innate immune system activation still represent a challenge toward the appraisal of the innate immunity role in the immune-mediated toxicities observed in gene therapy trials.

Innate immunity is the first barrier against pathogens as it mounts rapidly and does not require a specific adaptation to the pathogens. Innate immune response depends on the recognition of pathogen-associated molecular patterns by the pattern recognition receptors (PRRs) expressed by immune cells. The molecular recognition of viral nucleic acids, membrane glycoproteins, or even chemical messengers by PRRs leads to the nuclear translocation of Nuclear Factor κB (NF-κB) and Interferon-Regulatory Factor (IRF), transcription factors with a central role in the expression of pro-inflammatory cytokines, or type I interferons (IFNs), respectively (65).

In the context of rAAV-mediated gene transfer, preclinical studies supported the important role of type I IFNs in the induction of CD8^+^ T cell responses. In particular, blocking the activation of innate immune responses prevented both cytotoxic (66, 67) and humoral (52) anti-capsid responses *in vivo*.

Most of the data on the role of type I IFNs in rAAV vectors immunogenicity were obtained in the context of liver-targeted gene transfer. The liver represents a unique immunological environment, characterized by the presence of resident immune cells together with both specialized and non-specialized antigen presenting cells (APCs). In non-parenchymal liver cells, including Kupffer cells and liver sinusoidal endothelial cells (LSECs), innate immunity activation after liver gene transfer with rAAV mainly occurs through binding to TLR2 expressed on the cell surface (68). The rAAV double-stranded DNA genome, and in particular its unmethylated CpG motifs, may be recognized by the endosomal TLR9 in either Kupffer cells (69), peripheral plasmacytoid DCs (pDCs) (66, 70) or monocyte-derived DCs (71). TLR9 engagement was associated with enhanced activation of AAV-specific CD8^+^ T cell due to increased antigen presentation on class I major histocompatibility complex (MHC) (54, 66, 72).

An intriguing hypothesis is that, in addition to the vector DNA genome, double-stranded RNA (dsRNA) may participate to the induction of innate immunity to rAAV (73). According to this study, dsRNAs are produced by the promoter activity of the ITRs. Accumulation of dsRNAs would, in turn, stimulates the MDA5 sensor in human hepatocytes transduced with AAV, leading to the expression of type I IFNs. Interestingly, the blockade of MDA5 decreased the IFN response and improved transgene expression in transduced cells *in vitro* (73). Although this hypothesis is not yet supported by clinical data, it may explain why cellular responses are sometimes initiated weeks after vector administration in clinical trials, a timeframe that is consistent with the dsRNA synthesis *in vivo* (51, 74).

### Adaptive Immune System Activation Following rAAV Administration

The induction of an adaptive immune response requires a longer time than innate immunity and is considered as the second barrier toward pathogens. On the other hand, adaptive immune responses are antigen-specific and eliminate pathogens while generating an immunological memory.

T and B lymphocytes are activated after the molecular recognition of an antigen presented by APCs (75). After activation, lymphocytes expand and differentiate into effector cells and specifically inactivate or clear antigens through the induction of humoral or cytotoxic responses. When the levels of circulating antigen are reduced by the mounting immune response, memory T and B lymphocytes are generated and can respond to successive antigenic stimulation in a more efficient and faster manner (75). After rAAV vector administration, both transduced cells and professional APCs present capsid-derived epitopes to cytotoxic CD8^+^ T cells via MHC class I (50, 56, 66, 76). Activated CD8^+^ T cells may clear rAAV-transduced cells thus inducing inflammation in the target organ, affecting the gene transfer outcome (45, 51, 77, 78). The concurrent presentation of capsid-derived MHC class II epitopes by professional APCs activates CD4^+^ T helper cells, which facilitate humoral and cell-mediated immune responses (67). Indeed, experience from clinical trials indicate that rAAV vectors administration leads to the development of anti-AAV IgG and NAbs (42), likely preventing vector readministration. Administration of immunomodulatory regimens (79) or, B-cell depletion prior to gene transfer, (80) have been effective in blocking humoral immune responses to rAAV in the preclinical setting. To this aim rituximab in combination with rapamycin is currently being tested in humans as a strategy to enable vector re-dosing (NCT02240407).

Clinical experience indicates that, to some extent, rAAV vector immunogenicity is dose-dependent (81, 82). Low vector doses appear to be managed, in most cases, by short courses of corticosteroids or other mild immunosuppressive regimens with rescue of transgene expression (82). Accordingly, a dose-dependent increase in AAV-antigen presented on MHC class I together with higher CD8^+^ T cells activation was reported *in vitro* (76, 83). Nevertheless, deleterious effects of anti-capsid cellular response, and in particular the very slow onset of the T cell response, are not fully recapitulated by animal models. This has prevented the investigators to formulate predictive models of rAAV vector immunogenicity, and to some extent hindered the development of specific immunomodulatory protocols specific for gene transfer.

## Immune Responses Against the Transgene Product

A large part of the gene therapy strategies for monogenic diseases aim at the replacement of a mutated gene with a corrected copy to restore its function and correct the disease phenotype. The potential development of an immune response against the transgene is dependent on several variables, including the tissue targeted with gene transfer, the host genetic background, and the extent of the residual expression of the donated gene. Gene transfer in a context of missense mutations and residual endogenous expression of the full length protein is unlikely to induce anti-transgene immune responses, although some preclinical studies suggest that even single amino acid variations can be recognized by the host immune system (84). Conversely, gene transfer in the context of stop mutations with no residual protein expression, is potentially more likely to result in anti-transgene immunity due to the absence of central tolerance against the transgene product itself.

In the clinical setting, anti-transgene immune responses were documented, so far, in only few clinical trials, mostly after intramuscular delivery of rAAV vectors. In particular, evidence of T cell-mediated anti-transgene cytotoxic T cell responses was documented in a phase I/II trial of intramuscular gene transfer in Duchenne muscular dystrophy patients (85). In this trial, AAV-mediated transfer of a mini-dystrophin transgene resulted in poor expression and was associated with the development of T cell responses directed against transgene epitopes or, possibly, in the recall of pre-existing anti-dystrophin T cells response. Similarly, decreased transgene expression and transgene-specific cytotoxic T cells were reported after intramuscular delivery of alpha-1 antitrypsin with rAAV vector in one subject (86), although most of the clinical trial participants achieved long-term expression of the transgene (87). In other clinical trials, the impact of immune response on the treatment outcomes was less clear. Finally, in a phase I/II trial of intracranial delivery of a rAAV5 vector for mucopolysaccharidosis type IIIB, anti-transgene T cells were also reported (88). Taken together, the clinical data presented suggest that disease-specific conditions e.g., the ongoing inflammation in muscle dystrophies (85), are likely to increase transgene immunogenicity after gene transfer.

The apparent inconsistency between the potential immunogenicity of the transgene and the low number of actual reports of anti-transgene immunogenic responses could possibly be explained also by the fact that most of the clinical trials, so far, were performed: (i) in subjects already exposed to protein replacement therapy prior to gene transfer; (ii) in subjects with residual endogenous expression of the gene targeted with gene transfer; (iii) by gene transfer restricted to immune-privileged compartments like the eye, the liver or the brain; and (iv) when gene transfer was administered together with an immunomodulatory regimen.

One important determinant of anti-transgene immune responses is the tissue distribution of transgene expression. The selectivity of gene expression for a given target tissue is the result of the combined tropism of the AAV capsid, the route of vector administration and the specificity of the promoter included in the transgene expression cassette. In general, intramuscular administration and strong ubiquitous promoters are more likely to induce anti-transgene immune responses than systemic administration and tissue-specific promoters (89, 90). Another layer of complexity in the evaluation of the potential immunogenicity of gene transfer is that, in some tissues, such as muscle (91), the presence of inflammation due to the underlying disease might result in a higher risk of triggering transgene-directed immune responses. Conversely, in tissues that are defined as immune-privileged *per se* due to the presence of barriers that reduce antigen presentation and immune system cells trafficking (e.g., the eye or the nervous system) or to their particular immunological milieu (e.g., the liver), the overall risk of encountering anti-transgene immune responses is low.

The liver, due to the constant exposure to non-self antigens, has peculiar immunological properties that prevent uncontrolled immune activation. Several studies of gene transfer with rAAV in both small and large animals indicated that hepatocyte-restricted transgene expression induced a robust, antigen-specific peripheral tolerance (60, 92, 93). In animal models, liver-induced immunological tolerance has been exploited to counteract deleterious immune responses induced by gene transfer targeting more immunogenic tissues, such as the muscle (89, 94). The different antigen presenting cells (APCs) resident in the liver are involved in the tolerogenic effect after liver gene transfer. In particular, Kupffer cells, the macrophages resident in the liver, seem to have a less mature phenotype compared to other professional APCs (95, 96). This, together with the secretion of the anti-inflammatory cytokine IL-10 (97–99) by Kupffer cells leads to poor T cell-activation. Antigen presentation through MHC class I expressed onto hepatocytes has been associated with incomplete CD8^+^ T cell activation and increased exhaustion and apoptosis (89, 93, 94, 100–103). Liver sinusoidal endothelial cells (LSECs) can also act as professional APCs and promote tolerance through the induction of T regulatory cells (Tregs) (104, 105). Tregs play an essential role in tolerance induction after liver gene transfer as demonstrated by the increased transgene immunogenicity observed after Tregs depletion (60, 89, 92, 106). Consistently, increased Tregs expansion by rapamycin treatment, favored the induction of liver-mediated tolerance even in the presence of pre-existing anti-transgene immunity (107, 108). Other mechanisms like the induction of CD8^+^ regulatory T cells (109), the degradation of T cells in hepatocytes (110), and the CD4^+^ T cell anergy (111) were proposed in the establishment and maintenance of liver tolerance.

## Immune Responses to rAAV Vectors in Clinical Trials After Intravenous Infusion of rAAV Vectors

### Liver Gene Transfer – The Experience With Hemophilia B

The largest set of clinical data available on rAAV-mediated gene transfer for the treatment of liver diseases derives from hemophilia B studies. Hemophilia B is an ideal target for rAAV gene therapy for different reasons. First, the transgene, human coagulation factor IX (hFIX), can be expressed in a variety of tissues including muscle and liver, the latter being its natural site of synthesis, and low levels of transgene expression (around 5% of normal) are sufficient to greatly reduce the impact of the disease on the quality of life of the patients. Another important advantage is that hFIX is small and, based on the experience with protein replacement therapy, seems to have a relatively lower immunogenicity potential compared to, for example, human coagulation factor VIII. Finally, the disease is very well characterized, small and large animal models of hemophilia B are available, and methods and endpoints to evaluate the efficacy of a given treatment are well established.

The first demonstration that hFIX can be secreted by human hepatocytes following rAAV vector-mediated gene transfer was obtained in a seminal clinical trial where 7 subjects with severe hemophilia B received through the hepatic artery a single-stranded rAAV2 vector carrying the hFIX transgene under the control of a liver-specific promoter (45). The clinical trial was designed with three increasing dose cohorts of 8 × 10^10^ vg/kg, 4 × 10^11^ vg/kg, and 2 × 10^12^ vg/kg, respectively. Therapeutic levels of hFIX expression were reported only in the first patient who received the highest vector dose. However, differently from animal models that showed a stable expression of hFIX over time (51, 112), in humans, transgene expression started to decline 4 weeks after vector injection. This decline was associated with a self-limited increase in liver transaminases and the detection of circulating AAV-specific CD8^+^ T cells (45, 50). In a second patient dosed at the 2 × 10^12^ vg/kg dose, no transgene expression was observed possibly due to the presence of an anti-AAV2 pre-existing humoral immune response (45).

This first clinical trial demonstrated that the rAAV vectors were safe and efficacious in liver targeting, although transgene expression was only transient. Both small and large animal models used in preclinical research failed to predict this negative outcome linked to an anti-AAV capsid cellular response. Nevertheless, this clinical trial provided unique information for the future use of the rAAV technology for gene transfer in humans. A second clinical trial was then carried out using rAAV8 serotype for the expression of hFIX (81). The improved transduction of hepatocytes achieved with this serotype (113) was combined with a self-complementary genome and a codon-optimized transgene sequence to optimize the expression in the liver (114).

In this second trial, participants were screened for pre-existing humoral response against AAV8 and only seronegative patients were included. Three doses of the vector were infused through a peripheral vein ranging from 2 × 10^11^ vg/kg to 2 × 10^12^ vg/kg. Differently from the previous trial, hFIX expression was detectable and reached 1–4% of normal in the low and mid dose cohorts (81). At the highest dose, in the first subject dosed from this cohort, 8 weeks post-infusion the therapeutic hFIX levels (approximately 8–10% of normal) started to decline and, similarly to the previous trial, an elevation of liver enzymes and an increase in circulating capsid-specific T cells was detected. A tapering course of steroids was administered to control the liver enzyme elevation, which allowed for rescue of transgene expression (81). Of the additional participants enrolled in high dose cohort, a total of 6, 4 developed a transient transaminitis that rapidly resolved after transient treatment with prednisolone (74). In this study, in most participants, and in particular those from the high dose cohort, a significant reduction in annualized bleeding episodes in the absence of recombinant hFIX prophylaxis was reported (74) with a stable transgene expression documented for up to 10 years (115).

Results obtained in a third clinical trial for hemophilia B further support the role of the total capsid dose as a determinant of rAAV vector immunogenicity. Subjects seronegative for anti-AAV antibodies received a relatively low dose (5 × 10^11^ vg/kg) of an AAV vector expressing a hyperactive variant of hFIX (hFIX-R338L). At this dose, only 2 out of 10 participants had an elevation of liver enzymes, which was successfully controlled with corticosteroids. In this study rAAV administration resulted in therapeutic transgene expression in all enrolled subjects (78).

rAAV5 was also used to express hFIX in hepatocytes of hemophilia B patients. This vector, produced in a baculovirus system, was injected at doses up to 2 × 10^13^ vg/kg, in 10 hemophilia B patients (116). Therapeutic levels of hFIX were observed for most of the treated patients. ALT elevation, which was reported in 3 out of 10 patients, was neither associated with T cells activation detected by ELISPOT, nor correlated with the presence of preexisting NAbs, and was treated by corticosteroids with no measurable decrease in hFIX transgene expression. Similarly, in a clinical trial for hemophilia A, the infusion of up to 6 × 10^13^ vg/kg of a rAAV5 vector expressing human coagulation factor VIII resulted in ALT elevation in several enrolled subjects (117). The impact of liver enzyme elevation on transgene expression in this study is being debated, although a decrease in factor VIII levels has been detected in several participants followed at long-term (118).

Corticosteroid treatment given in response to ALT elevation usually controlled the anti-AAV vector immune response and stabilized transgene expression. However, in some cases this approach has failed to control the anti-capsid immune responses. One example is a clinical trial in which 7 hemophilia B subjects received a scAAV8 vector expressing the hyperactive hFIX-R338L variant (119) at doses ranging from 2 × 10^11^ vg/kg to 3 × 10^12^ vg/kg (NCT01687608). Sustained transgene expression (hFIX activity of about 20%) was reached in only one participant, while all other patients enrolled in the trial had either no expression or lost transgene expression despite corticosteroid treatment within 5 to 11 weeks post vector infusion without any evidence of anti-hFIX antibodies formation (120). Vector immunogenicity was possibly dependent on the elevated CpG content of the transgene expression cassette. Indeed, transduction of primary human liver cells with this vector induced the secretion of more Th1-oriented chemokines compared to a CpG-null version of the same vector (121). In a second clinical trial, rAAVRh10 serotype expressing hFIX was administered to six seronegative hemophilia B patients at doses of 1.6 × 10^12^ and 5 × 10^12^ vg/kg (122). A transient expression of hFIX was reported also in this study. After vector administration, 5 out of 6 injected individuals had ALT elevation associated with loss of the transgene expression despite corticosteroid treatment. Four of them demonstrated low anti-capsid and anti-hFIX response as measured by IFN-γ ELISPOT assay, possibly reflecting the high doses of corticosteroids they received. Subject 6 had a higher ALT elevation, associated with a strong CD4^+^ IL-2^+^ IFN-γ^+^ T cell response against an epitope spanning the hFIX mutation, and increased inflammatory cytokines in the serum (123), however, no anti-hFIX humoral immune response was documented in this subject.

Taken together, the clinical experience accumulated with clinical trials for Hemophilia B indicate that mild immune responses to the vector may clear transgene expression from the liver. These immune responses are controlled by corticosteroid treatment in most of the cases. However, transient expression of the hFIX transgene was observed regardless of corticosteroid treatment in some clinical trials, potentially due to a higher intrinsic immunogenicity of the vectors used.

### AAV Gene Transfer for Neuromuscular Diseases

Neuromuscular diseases of genetic origin are a multitude of diseases that are heterogeneous in terms of pathophysiology, tissues involved, age of onset, and clinical manifestations. Clinical experience accumulated on gene replacement with rAAV vectors indicates that regardless of the disease, muscle or central nervous system targeting requires high vector doses in the range of 1 × 10^14^ vg/kg. In recent years, the improvements in large-scale rAAV vector manufacturing allowed for delivery of the large doses of AAV vectors needed in patients with neuromuscular diseases.

One of the most exciting results in the field of rAAV-mediated gene transfer was the recent approval of Zolgensma (Avexis, Novartis) for the treatment of spinal muscular atrophy (SMA) type I (124). The efficacy of this drug was initially proved in a pivotal clinical trial involving 15 patients with SMA type I (125). A rAAV9 expressing the SMN1 gene under the control of a ubiquitous promoter was infused intravenously at doses of 6.7 × 10^13^ and 2.0 × 10^14^ vg/kg. In the first patient dosed at the low vector dose, a robust ALT elevation (31 times above the upper limit of a normal range) was reported although it was controlled by corticosteroids. After this first observation, prophylactic corticosteroids treatment was applied (30 days, starting 1 day before vector infusion) and ALT elevation was reported only in 3 patients within the high dose group (125). At the same time, high percentage of capsid-specific T cells were detected in peripheral blood by IFN-γ ELISPOT (126). However, neither the ALT elevation nor the presence of capsid-specific T cells was associated with reduced vector efficacy. The excellent efficacy profile and the mild adverse events reported in this first clinical trials were confirmed at long-term (125, 127, 128) and also in a phase III trial (ClinicalTrials.gov: NCT03306277). Based on these results, a dose-finding clinical trial was launched to evaluate the safety and efficacy of intrathecal administration of Zolgensma in SMA type 2 patients (ClinicalTrials.gov: NCT03381729). So far, 31 patients received the treatment at the three vector doses ranging from 6 × 10^13^ to 2.4 × 10^14^ total vector genomes (129). Results of the first two dose cohorts indicated an amelioration of the motor function with mild adverse events. Despite corticosteroids treatment, elevated ALT and AST were reported in one patient and were considered related to the treatment. However, this trial was placed on partial hold by the FDA after the detection of possible toxicity in dorsal root ganglia neurons in two independent preclinical studies performed in large animals (130, 131). Importantly, this unexpected toxicity was never reported in patients that received the same vector by peripheral vein (125) or in a different trial of rAAV gene transfer for giant axonal neuropathy (ClinicalTrials.gov: NCT02362438) in which intrathecal delivery of rAAV9 was associated with transient corticosteroids and a longer course of tacrolimus and rapamycin.

Additional information on the administration of high doses of rAAV in pediatric patients derives from a Phase I/II clinical trial for X-linked myotubular myopathy (MTM, ClinicalTrials.gov: NCT03199469) and three studies for Duchenne muscular dystrophy (DMD, NCT03375164, NCT03362502, NCT03368742). In the MTM study, doses up to 3 × 10^14^ vg/kg of a rAAV8 vector expressing the Mtm1 transgene under the control of a muscle-specific promoter were administered to patients less than 5 years old (132). Clinically meaningful improvement together with robust protein expression and recovered histology were reported. In this study, a prophylactic regimen of corticosteroid was applied. Although the vector was generally well tolerated, increased liver enzymes, creatinine kinase and troponin were reported. Both anti-transgene and anti-capsid immune responses were detected after vector administration, although it is unclear whether there is any correlation with the clinical outcomes. Further studies and long-term follow-up of participants will help to elucidate the relevance of the measured immune responses.

Variable outcomes across trials were reported in the context of systemic rAAV vector delivery for DMD. One clinical trial used a rAAVrh74 vector to express a micro-dystrophin transgene with the MHCK7 muscle specific promoter (ClinicalTrials.gov: NCT03375164). In this trial, 3 out of 4 DMD patients who received a dose of 2 × 10^14^ vg/kg had elevated liver enzymes, which were controlled by increasing corticosteroid dose (133). So far, no data was provided by the sponsor on specific responses against the capsid or the transgene, although sustained transgene expression, together with amelioration of circulating creatine kinase levels (133), suggest no impact of immune responses on muscle transduction. A randomized, double blind, and placebo-controlled study was recently opened to strengthen the data of this pilot study (ClinicalTrials.gov: NCT03769116). Two weeks after vector injection, a case of rhabdomyolysis was reported in a participant from this second study. However, the study drug safety monitoring board reviewed the data and recommended the study to continue, thus suggesting that the adverse event was possibly unrelated to the investigational product.

Two additional studies of systemic rAAV vector delivery for DMD presented a more complex clinical picture. In the first one, a rAAV9 vector expressing a micro-dystrophin under the control of a muscle-specific promoter was infused intravenously in 6 adolescent patients at two doses, 5 × 10^13^ and 2 × 10^14^ vg/kg (ClinicalTrials.gov: NCT03368742). Despite microdystrophin expression, this trial was placed on hold because 2 patients, one in the low dose and the second in the high dose cohort developed acute kidney injury and activation of the complement system with signs of cardiopulmonary decline (134). In a second trial for DMD, 1 × 10^14^ vg/kg or 3 × 10^14^ vg/kg of an rAAV9 vector expressing a mini-dystrophin under the control of muscle-specific promoter was injected in 6 adolescent patients from 6 to 12 years of age (ClinicalTrials.gov: NCT03362502). Mini-dystrophin expression levels above 20% of wild-type were observed at 2 months post vector infusion, while activation of the immune system, as measured by neutralizing antibody levels and T-cell responses with ELISPOT, was documented in all the participants (135). As in the previous clinical trial, one of the participants showed symptoms of complement activation and acute kidney injury that required a treatment with a complement inhibitor (Eculizumab). Importantly, the sponsor observed that the activation of the complement was associated with a rapid antibody response against the vector (135).

The data obtained from clinical trials in neuromuscular diseases support the concept that high doses of rAAV vectors are in general well tolerated and have the potential to treat rare genetic diseases affecting muscle or central nervous system, particularly when these high doses are administered during early childhood. However, in adolescent patients, complement activation, possibly due to exaggerated anti-vector immune responses may represent a limitation to the application of gene therapy at such doses. It should be noted that this hypersensitivity to rAAV was identified in DMD patients that, due to the ongoing muscle degeneration and the underlying inflammation, have a peculiar immunological environment that tends to exacerbate immune responses (91). The disease-specificity of these responses is possibly supported by the fact that in a clinical trial for Limb-girdle muscular dystrophy type E, no instances of immune mediated toxicities were reported despite the use of a similar vector at comparable doses. Other triggering factors are also being considered to explain these emerging clinical findings, such as the age of the subjects enrolled, the different vectors used in the studies, and eventually contaminants derived from the different processes used to manufacture the clinical lots of rAAVs used in the studies. Larger studies will help to better define the determinants of the immunotoxicities observed in those trials.

## Conclusion

In recent years, we have accumulated significant clinical experience with AAV vectors. While the study of immune responses in AAV trials has resulted in important advances for the field, a lot more needs to be done to provide a clear picture of the complex interactions of AAV with the host immune system in the different clinical settings of gene transfer. Of note, relatively little is understood on the host- and vector- dependent factors influencing the development of cytotoxic immune responses leading to poor gene therapy outcomes in terms of duration of transgene expression. To this end, one limitation of the current immunomonitoring methods is that they rely on the *in vitro* testing of immune cells isolated from peripheral blood vs. lymphocytes infiltrating the peripheral tissues transduced with rAAV vectors. A distinct activation profile of tissue-resident cells, and the variability associated to PBMCs collection and testing across clinical trials (i.e., the lack of standardization of assays used for immunomonitoring), may explain the poor correlation between IFN-γ ELISPOT and immunogenicity outcomes observed in some clinical trials. Additionally, the monitoring of cytokines other than IFN-γ may be helpful in more effectively monitor T cell activation following rAAV vector administration (52) (Figure 3). The relatively low number of subjects enrolled in each trial also represents a potential limitation to the study of immune responses after gene transfer, although the analysis of the collective results in the clinic, along with preclinical studies, has begun to highlight some of the determinants of vector immunogenicity (e.g., the ability of rAAV vectors to activate innate immunity). A more systematic and standardized approach to immunomonitoring in rAAV trials may help further boost our knowledge on vector immunogenicity in humans, particularly for what concerns the understanding of the importance of the disease-specific immune context of gene transfer. This will be key to devise strategies aimed at reliably achieving safe and long-lasting therapeutic efficacy following rAAV vector delivery.

## Author Contributions

GR, D-AG, and FM wrote the manuscript.

## Conflict of Interest

FM was employed by Spark Therapeutics, a Roche company. FM and GR are inventors in patents related to AAV gene therapy and the control of immune responses against AAV vectors. The remaining author declares that the research was conducted in the absence of any commercial or financial relationships that could be construed as a potential conflict of interest.
